# Static and Dynamical Quantum Studies of CX_3_-AlX_2_ and CSiX_3_-BX_2_ (X = F, Cl, Br) Complexes with Hydrocyanic Acid: Unusual Behavior of Strong π-Hole at Triel Center

**DOI:** 10.3390/ijms24097881

**Published:** 2023-04-26

**Authors:** Mariusz Michalczyk, Kamil Wojtkowiak, Jarosław J. Panek, Aneta Jezierska, Wiktor Zierkiewicz

**Affiliations:** 1 Faculty of Chemistry, Wrocław University of Science and Technology, Wybrzeże Wyspiańskiego 27, 50-370 Wrocław, Poland; 2 Faculty of Chemistry, University of Wrocław, ul. F. Joliot-Curie 14, 50-383 Wrocław, Poland; kamil.wojtkowiak@chem.uni.wroc.pl (K.W.); jaroslaw.panek@chem.uni.wroc.pl (J.J.P.); aneta.jezierska@chem.uni.wroc.pl (A.J.)

**Keywords:** molecular electrostatic potential, triel bond, QTAIM, NCI, SAPT, BOMD

## Abstract

The set of TX_3_-TrX_2_ (T = C, Si, Ge; Tr = B, Al, Ga; X = F, Cl, Br) molecules offers a rather unique opportunity to study both σ-hole and π-hole dimerization on the tetrel and triel ends, respectively. According to the molecular electrostatic potential (MEP) distribution, the π-hole extrema (acidic sites) were more intense than their σ-hole counterparts. The molecules owning the most (CX_3_-AlX_2_) and least (SiX_3_-BX_2_) intense π-holes were chosen to evaluate their capacities to attract one and two HCN molecules (Lewis bases). We discovered that the energetic characteristics of π-hole dimers severely conflict with the monomers MEP pattern since the weakest π-hole monomer forms a dimer characterized by interaction energy compared to those created by the monomers with noticeably greater power in the π-hole region. This outcome is due to the deformation of the weakest π-hole donor. Furthermore, the MEP analysis for monomers in the geometry of respective dimers revealed a “residual π-hole” site that was able to drive second ligand attachment, giving rise to the two “unusual trimers” examined further by the NCI and QTAIM analyses. Apart from them, the π-hole/π-hole and σ-hole/π-hole trimers have also been obtained throughout this study and described using energetic and geometric parameters. The SAPT approach revealed details of the bonding in one of the “unusual trimers”. Finally, Born-Oppenheimer Molecular Dynamics (BOMD) simulations were carried out to investigate the time evolution of the interatomic distances of the studied complexes as well as their stability.

## 1. Introduction

The groundbreaking discovery of σ- and π-hole electron-deficient regions [1] on certain parts of single molecules opened a wide discussion about potentially possible new junctions between particular entities. The origin of the contacts that were earlier observed in the solid state [2,3,4,5,6,7,8] became more understandable, which triggered searching for and examining (both theoretically and experimentally) new synthons grounded on the electron density donor-acceptor relations. The literature is crowded with examples of the role of σ-hole and π-hole interactions in steering the structural arrangement in crystal structures and acting as driving forces for the self-assembling of supramolecular architectures, even if only the last two years are considered [9,10,11,12,13,14,15,16,17,18,19,20,21,22].

Triel [23,24,25,26,27,28] and tetrel [29,30,31,32,33,34,35] bonds (abbreviated as TB and TrB, respectively) are classified as relatively new members of the family of σ/π-hole interactions. Especially the latter is found in many studies devoted to the crystal engineering aspects [30,31,33,36,37,38,39,40,41,42,43,44,45,46,47]. The term “σ-hole” was introduced in the middle of the first decade of the 21st century [48,49] and refers to the area connected to an atom that is covalently bonded to the electron-withdrawing substituent. Such an association dilutes the electron density on the outer surface of that atom. The measurable effect is a significant growth of the electrostatic potential on the outer portion of the atom surface [50,51]. The ETS-NOCV (*natural orbitals for chemical valence*) analysis, which assesses the contributions to deformation density arising from the electron charge transfer channels, can be alternatively used to produce another description of the σ-hole area [52]. Thus, an affected fragment acquires the properties of Lewis acid, attracting centers with a large electron density, such as, for example, electron lone pairs. As a corollary to this, the scientific literature has witnessed a fiery growth of studies dedicated to new noncovalent interactions, beginning with those concerned with the halogen bond [53,54,55,56]. The mechanism of forming complexes through tetrel, triel, and other bonds of this group is commonly rationalized on the basis of the Lewis acid⋯Lewis base electrostatic interaction heavily doped by the orbital interaction and dispersion [29,57]. However, there are differences in details between them. Namely, the tetrel bond is mainly powered by the σ-hole region, while the triel bond is powered by the π-hole one. This is due to the fact that tetravalent tetrel atoms produce overlapping σ-holes on the extension of four T-R bond axes (where R is the electron-withdrawing agent), while trivalent triels organized in planar TR_3_ molecules are able to generate two π-holes located symmetrically, perpendicular to the molecular plane. Their position is then the largest difference between them, which obviously influences the geometry of complexes formed via tetrel or triel bonds. So far, it has been shown that the TrR_3_/TR_4_ (Tr = Al, Ga, In; T = Si, Ge, Sn) monomers undergo significant structural deformations throughout the complexation with the N-bases such as HCN, NH_3_, or CN [58]. Triel and tetrel bonds in these complexes were scrutinized by theoretical methods, and the results demonstrated that the formation of tetrel and triel bonds forces the internal rearrangement of the Lewis acid molecule due to the strong electrostatic attraction between the units. Triel-bonded complexes adopted tetrahedral geometry, while those with tetrel atoms distorted to a trigonal pyramid shape. The quantitative description of these processes indicated that the intensification of π-hole during complexation is from 13 to 63% in comparison with its value in the optimized monomer, and the amount of energetic cost required for adjusting the geometry of Lewis acid to that in the complex is up to even 42 kcal/mol [58]. As far as our knowledge extends, there are no further reports of systems that contain concurrently triel and tetrel atoms and, as a consequence of that, two separate fragments: σ-hole and π-hole on the same molecule.

**Figure 1:**
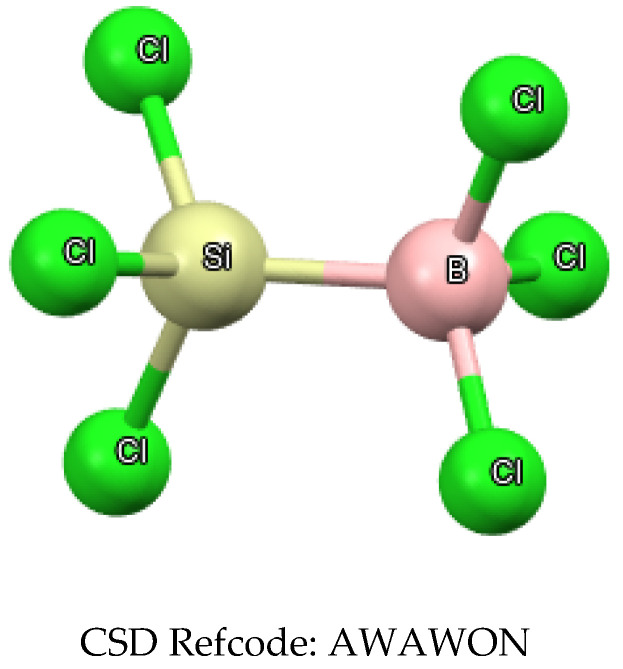
Sample from CSD survey of fragments of crystal structure [60] representing similar architecture to those studied within this work.

Within the current work, we aim to investigate closer the distribution of the molecular electrostatic potential (MEP) on the triel and tetrel-containing molecules, characterized in their initial state by two kinds of holes: σ (at the tetrel atom) and π (at the triel center), and further the ability to form heteropolymers between these Lewis acids equipped with the hole region and an approaching nucleophile. We focus more specifically on the π-holes of different depths in the chosen set of monomers and their behavior during the complexation reaction with the Lewis base (hydrogen cyanide, HCN). The simplicity of this small ligand guarantees that there will be no interfering interactions in the studied systems. Our research is guided by the following questions: Does the heterodimerization through weak and strong π-holes differ? How does the first complexation affect the distribution of the MEP on Lewis acid in the geometry of the dimer? Does the attachment of the first nucleophile allow for the incorporation of the next one? If so, how would the second complexation occur? Figure 1 displays the real anionic structure from the CSD search [59], which is the representation of the discussed issue (a complete list is provided in the SI, see Figure S1). In each case, the triel atom is tricoordinated, but one of the ligands attached to the triel is directed into its π-hole. Therefore, this situation mimics the following: the dicoordinated triel center is attacked by the Lewis base through the nucleophilic site, thereby forming the triel⋯halogen dimer. In the wake of that, we built the theoretical models of monomers belonging to the series TX_3_-TrX_2_ (Tr = B, Al, Ga; T = C, Si, Ge), and further, we selected the most and least powerful monomers CX_3_-AlX_2_ and CSiX_3_-BX_2_ (X = F, Cl, Br) in terms of their π-hole magnitude to study their heterodimers and trimers with HCN as the Lewis base. To this end, we used quantum chemical protocols such as MEP, QTAIM, NCI, or SAPT along with the ab initio molecular dynamics (AIMD) simulation. An application of the last method gives an insight into the time-evolution of selected metric parameters and, consequently, allows us to estimate the stability of complexes and track how the strength of noncovalent interactions changes. We believe that our work will expand our knowledge about the abovementioned types of noncovalent interactions and introduce novel data concerning the interaction scheme when more than one acidic binding site is available on the electron density-acceptor molecule.

## 2. Results and Discussion

### 2.1. Monomers

**Scheme 1:**
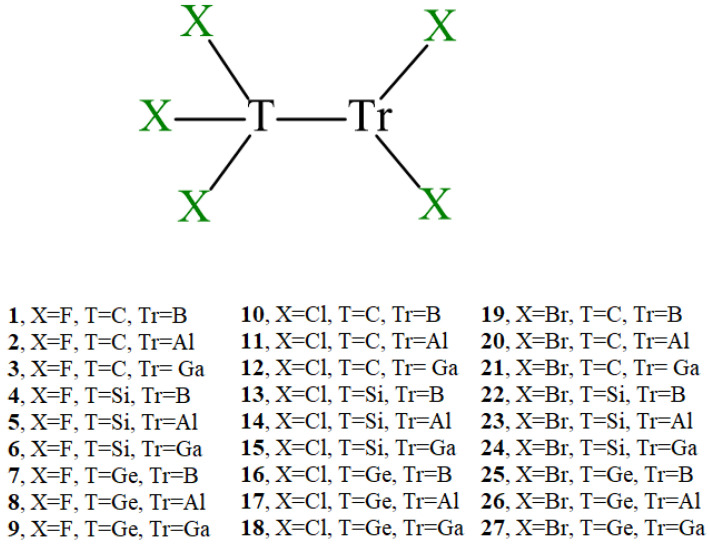
Schematic representation of studied monomers: T = C, Si, Ge; Tr = B, Al, Ga; X = F, Cl, Br.

The chosen set of monomers is represented in Scheme 1. The TX_3_-TrX_2_ (Tr = B, Al, Ga; T = C, Si, Ge) monomers were fully optimized and evaluated in view of the MEP extrema on their surfaces. The geometry of selected monomers allowed us to suppose that they are characterized by two types of electron-depleted regions: σ-holes on the tetrel (T) center and π-holes on the triel (Tr) atoms. In the case of six monomers where T = C and X = Cl, Br, the CX_3_ group is slightly pivoted versus the TrX_2_ group, which results in breaking the symmetry of the entire molecule and, as a consequence, unevenness of the potentials (V_s,max_ values) of the π-holes. The effect of the CX_3_ moiety motion on the π-hole magnitude is highlighted in the first rows of Tables S2 and S3. One of the π-holes in the specific instance of CCl_3_-BCl_2_ was even two times stronger than the other. The global magnitude difference between these holes in those six cases was in the range of 15 to 30 kcal/mol. In Table 1 there are collected only the strongest π-hole maxima found at the triel atoms.

In Table 1, the MEP extrema for 27 monomers are collected. In the table above, the values of weaker V_s,max_ extrema (σ-holes) are omitted (they are given in equivalent Tables S1–S3 in the SI). One can see a tendency in these results that the magnitude of the hole decreases in line with the X substituent in order: F > Cl > Br. The π-hole strength is also related to the nature of the Tr atom—one can see that the Al atom’s presence within the monomer produces a stronger hole, followed by Ga and B. For a given X atom, the combination of triel and tetrel atoms such as Al and C guarantees the boldest π-hole, while B and Si atoms guarantee the weakest one. These edge cases are indicated in orange in Table 1, and they were nominated for further study (dimerization with Lewis bases). The strongest π-holes overrun 100 kcal/mol (up to nearly 112 kcal/mol). These regions of similar magnitude were observed in earlier reports [23,61,62]. The softest π-holes are 27–35 kcal/mol, which agrees with the inspection made by Yang et al., where the π-hole at BBr_3_ [62,63] was 31 kcal/mol.

**Figure 2:**
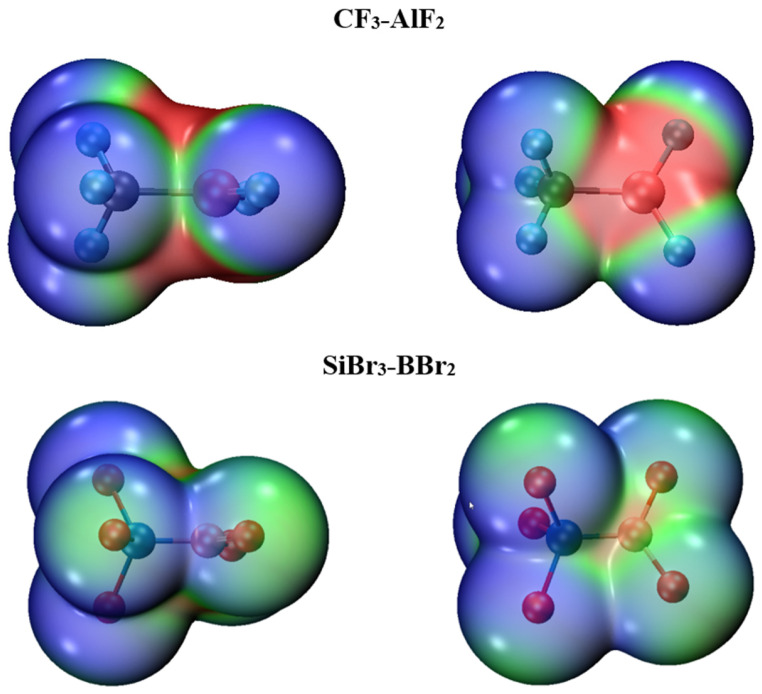
MEPs of CF_3_-AlF_2_ (**top**) and SiBr_3_-BBr_2_ (**bottom**) in two projections for the monomers with the most and least intense π-holes from all investigated, respectively. Isosurface 0.001: au; MEP scale: −0.01 (blue) to 0.05 (red) au.

The distribution of MEP is presented in Figure 2 for the outermost cases concerning monomers **2** and **22**. From that figure, one can see the difference in the size of the spilled positive center of the molecular electrostatic potential. In the (**2**)CF_3_-AlF_2_ case, the π-hole region (in red) is broad, while in the case of (**22**)SiBr_3_-BBr_2_, it is only a small speck. When one inspects the composition of monomers having the most and least intense π-holes, it may be concluded that the strongest hole is produced for the monomer consisting of atoms of the highest electronegativity (C—2.55) and the lowest one (Al—1.61). Such a connection causes a more efficient withdrawal of electron density from the triel center and the appearance of a larger π-hole (the difference in electronegativities is 0.94) at the triel atom. On the other hand, the monomer with the lowest π-hole is built from the Si and B atoms, then the least electronegative tetrel and most electronegative triel, respectively. The difference between them is only 0.14, and the triel is the one with higher electronegativity in this case. Therefore, the effectiveness of the depletion of electron density at the B atom attached to silicon has to be significantly worse than in the case of C-Al linking. Moreover, the halogen substituents additionally exacerbate these differences. Monomer **2** has fluorine substituents, which are more electron-withdrawing than bromine atoms in monomer **22**. As an effect, we have obtained two monomers with π-holes that differ by roughly 85 kcal/mol in their magnitudes.

### 2.2. Dimers

**Figure 3:**
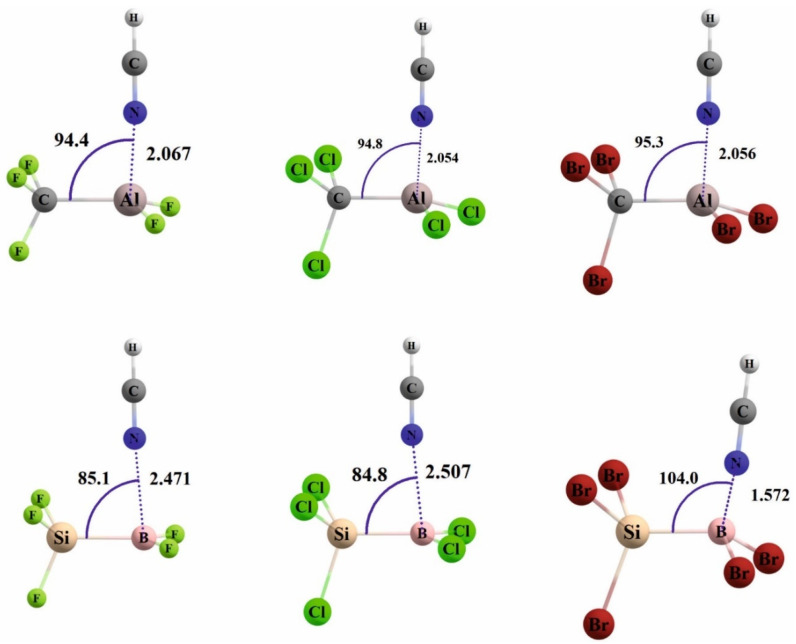
Geometry of dimers with HCN studied in this work. Distances in Å, angles in degrees.

In order to check the dimerization ability in opposite environments connected with the acidic potential of the binding site, the monomers with bordering potential values (strongest and weakest within the groups with different halogen atoms) were selected for this part of the study, namely the **2**, **4**, **11**, **13**, **20,** and **22** monomers. The geometries of binary complexes of selected monomers with HCN have been fully optimized. During the modeling, the HCN molecule was attached to the most intense extremum (π-hole site; see Table 1). The alignment of the obtained dimers is staged in Figure 3, whereas the crucial geometric and energetic descriptors, along with the MEP extrema of π-hole for monomers, are summarized in Table 2.

According to these results, it is quite surprising that for dyads with X = Br, the complex **22** (SiBr_3_-BBr_2_⋯NCH) has more favorable interaction energy (−28.72 versus −27.48) than the complex **20** (CBr_3_-AlBr_2_⋯NCH), despite the fact that the latter has an EP maximum of exactly 44 kcal/mol larger than the former. For some reason, the Lewis base is able to attach to the π-hole at a much shorter distance in SiBr_3_-BBr_2_ than in CBr_3_-AlBr_2_. Therefore, we checked the two other monomers with boron with other tetrels (C and Ge, not included in Table 2). The electrostatic potential (EP) maxima of monomers were very similar to those in SiBr_3_-BBr_2_ (see Table 1). The obtained interaction energies were also surprising, as they are comparable to those for SiBr_3_-BBr_2_ and larger than those for CBr_3_-AlBr_2_, which should be the most powerful. In other words, the interaction energy of complexes does not correlate with the MEP prediction. This is not the case when X = F or X = Cl (complexes in duels **2** and **4** or **11** and **13**, respectively). In those cases, the complexes with monomers of lesser σ-holes are indeed energetically weaker. The deformation of Lewis acid occurring during the complexation is displayed in the last column of Table 2. This effect is responsible for the puzzling outcomes described above. It must be remembered that the deformation energy is the cost required for adjusting the geometry of monomers to that observed in the complex. From another perspective, it is simply the difference between binding and interaction energy. The former refers to the fully optimized isolated monomers, while the latter refers to the monomers in the geometry of complexes. The deformation energies for unusually stable complexes are about 16.6 kcal/mol, while the remaining ones (those in line with the MEP forecast) have deformation energies of less than 5 kcal/mol. The contribution to this deformation is almost fully assigned to the distortion of Lewis acids geometry since the HCN distortion is in each case less than 0.5 kcal/mol. The deformation effect manifests itself in the value of the torsional T-Tr-X-X angle and, more precisely, its deviation from 180°. For **22** dimers, this difference is the largest and is 51.7° (the value of the Si-B-Br-Br angle is 128.3°). On the other hand, for the system with the lowest deformation energy, the value of the analogous dihedral angle is 171.8° (the deviation from 180° is only 8.2°). In addition, the planar Si-B⋯N angle in complex **22** is higher by 10 to 20° than the corresponding ones in the remaining dyads, which suggests that the arriving Lewis base is able to expand the area available for an effective attack. The final aftermath of deformation on monomer **22** makes the Lewis acid molecule more accessible to the incoming nucleophile, which is manifested not only in the ostensibly overvalued interaction energy but also in the B⋯N distance, which is significantly shorter than in the remaining complexes (1.572 Å versus 2.0–2.5 Å).

Additionally, to check for this apparent anomaly in different systems, we performed optimization and interaction energy calculations for dimerization with other Lewis bases: neutral ammonia and the CN^−^ anion. Results are gathered in Table 3. The selected Lewis bases are characterized by a negative potential higher than HCN. The neutral one, the ammonia molecule, has a V_s,min_ value of −37.7 kcal/mol, while for the cyanide anion, it is −137.7 kcal/mol. When one confronts the energies of complexes with ammonia and CN^−^ with the MEP values of bare Lewis acid molecules collected in Table 1, we can see once again that complexes with monomer **22** have interaction energies boosted by the geometry distortion, which makes them the most tightly bonded dimers of all studied, which is contrary to the MEP extrema obtained for isolated monomers.

**Figure 4:**
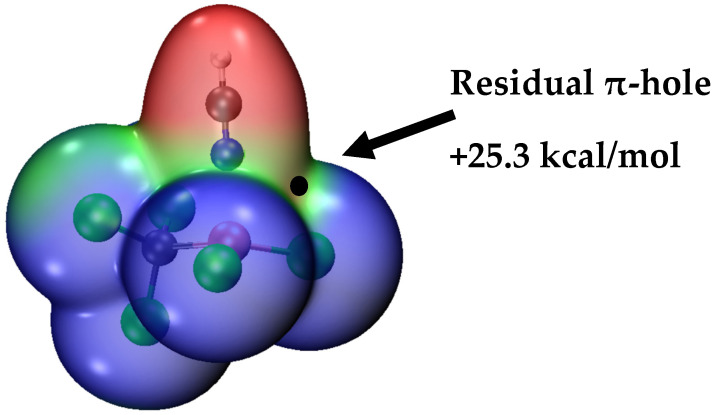
MEP of CCl_3_-AlCl_2_ complex with HCN. Black dot indicates the newly emerged maximum on the electrostatic potential surface. Isosurface 0.001 au; MEP scale: −0.01 to 0.03 au.

Furthermore, we have calculated MEP for all studied dimers, including those with NH_3_ and CN^−^ molecules (the results are displayed in Table 4). Attaching any nucleophile to a more intense π-hole leaves the symmetric π-hole in 16 out of 18 cases. For 8 dimers, the residing maximum has now a negative value (including all dyads with cyanide anion), and the remaining ones are featured by a second π-hole of magnitude ranging from 13 to 59 kcal/mol. What is perhaps most interesting from these results is that there were detected additional extrema representing the third maximum (“residual π-hole”) that emerged after attaching HCN to three monomers (**2**, **11**, and **20**), such that those with V_s,max_ at the Tr atom were the greatest (these V_s,max_ values are in Table 5 bolded in parentheses). Thus, one can say that inserting HCN in these cases did not completely saturate the acidic site but released the narrow area of positive MEP, which can potentially attract subsequent Lewis base molecules (see Figure 4). The magnitude of this new acidic site is in the range of 22.6 to 25.3 kcal/mol. It is interesting to compare the value of this new hole with the value of the MEP extremum of the second, classic π-hole. While for complex **2** with fluorine, the residual hole is clearly weaker in strength, for complex **11** with chlorine, both are comparable and in the case of complex **20** with bromine, the residual π-hole is even greater than the “normal” π-hole. In the case of Lewis bases other than HCN and complexes with HCN where triel-tetrel monomers had weaker MEP extrema (**4**, **13**, **22**), there are no residual π-holes (the π-hole site is completely saturated). As a result, we have decided to look closer at the complexes with HCN. The MEP map below illustrates the location of a new hole after the complexation of monomer **11** with HCN.

**Figure 5:**
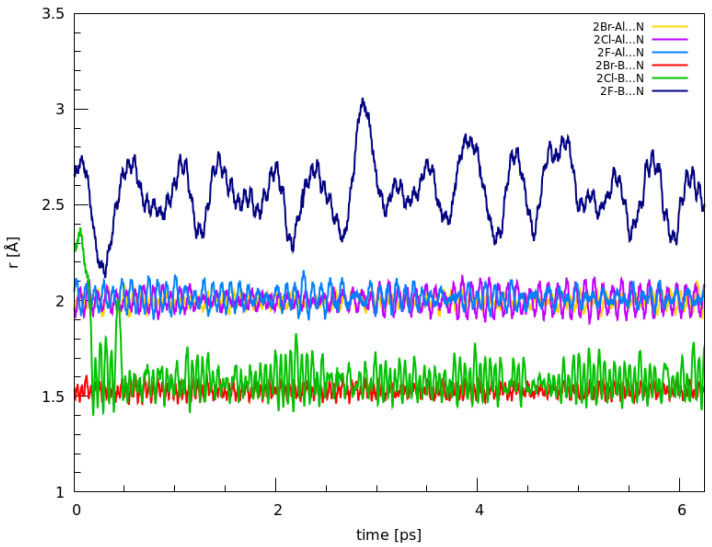
Time-evolution of metric parameters between the interacting pairs. Calculations performed at the PBE0-D3BJ/TZVP-MOLOPT-GTH level of theory.

We have recently used car-parrinello and path integral molecular dynamics (CPMD and PIMD) to investigate complexes containing diverse networks of intermolecular hydrogen bonds [64]. In the current study, Born-Oppenheimer molecular dynamics (BOMD) simulations in the NVE ensemble allowed for the estimation of the stability of the examined complexes. The results concerning the dimers (N…Tr distances) are presented in Figure 5 and Figure S2. It can be observed that in both cases, the results for SiBr_3_-BBr_2_⋯NCH and SiCl_3_-BCl_2_⋯NCH are similar, and the values of N…Tr distances assumed the smallest values among studied complexes and were equal to ca. 1.50–1.55 Å and 1.60–1.70 Å, respectively.

These values are reminiscent of the experimental B-N bond length of 1.673 Å in the archetypal BF_3_-NH_3_ adduct [65]. In the case of the SiF_3_-BF_2_⋯NCH dimer, it can be noted that the N…Tr distance oscillated around ca. 2.60 Å and 2.50 Å (for PBE and PBE0, respectively). Careful analysis of Figure 5 shows that a series is formed: the fluorine-bearing monomer keeps the HCN monomer at a distance, the Cl-containing monomer keeps the 2.5 Å distance for a short while (0.2 ps), and then the bond is shortened, while the SiBr_3_-BBr_2_ is strongly bound to the HCN from the very start. This suggests that the barrier separating the weaker non-covalent complex from the stronger adduct is highest for the SiF_3_-BF_2_⋯NCH, and the role of the halogen atom is significant. On the contrary, the values obtained for N…Tr distance for the dimers with T = C, Tr = Al, and X = F, Cl, and Br oscillated in a less pronounced way around 2.00 Å for each. As a final note, we would like to emphasize that the use of the hybrid exchange-correlation functional (PBE0) did not change the outcome of the BOMD simulation for the dimers of interest. As will be observed, the results for the trimers do not lead to the same conclusion.

### 2.3. Trimers

As the next step, trimers with two HCN molecules attached to the triel-tetrel centers were fully optimized. The resulting complexes corresponded to three groups: (I) trimers where the second HCN is aligned to the π-hole on the other side of the triel atom. As a result, in this scenario, two HCNs are joined electrophile opposite. This structural type will be henceforth called π-hole/π-hole; (II) trimers where the second HCN is glued to the residual π-hole (indicated in Figure 4—we baptized them as the “unusual trimers”); (III) trimers where one HCN is attached to π-hole (triel bond) and the second attacks the σ-hole at the tetrel atom (tetrel bond). These are labeled π-hole/σ-hole trimers. The summary of which trimers were found and which were not is given in Table S4.

#### 2.3.1. π-Hole/π-Hole Trimers

**Figure 6:**
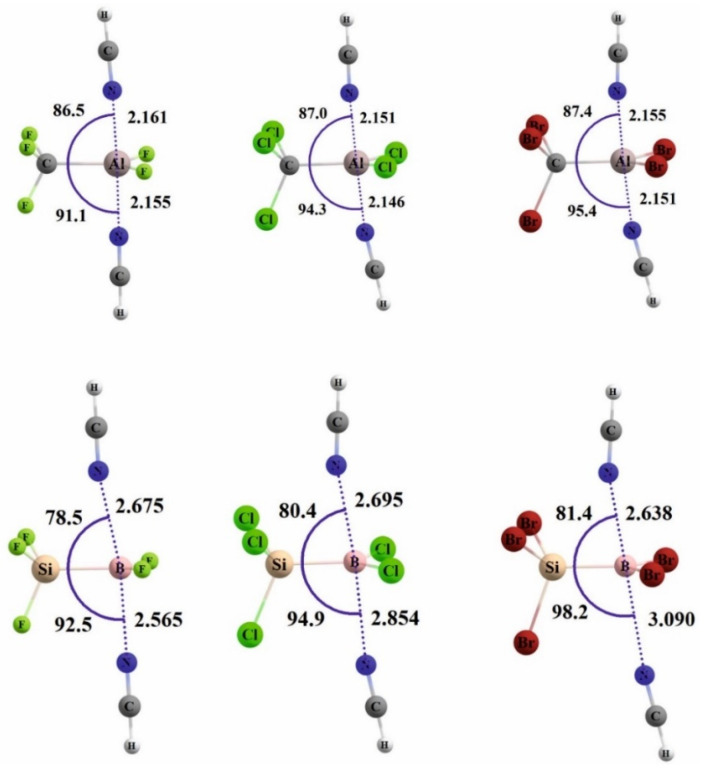
Optimized structures of trimers obtained by attaching the second HCN molecule to the second π-hole site of TX_3_-TrX_2_⋯NCH dimers (π-hole/π-hole trimers).

The π-π complexes were achieved for all six options, even for dimer **22**, where the second π-hole was of negative sign. The geometries of these trimers are stored in Figure 6.

The full characteristics of the π-hole/π-hole trimers are presented in Table 5. Interaction energies are highest for this group among all triads, as they range from −1.6 to roughly −17.5 kcal/mol. The specific values for a given trimer were counted for two variants: dimer LA molecule + first HCN with a single second HCN molecule (top value) and dimer LA molecule + second HCN with a single first HCN ligand (bottom value). Such construction always creates two separate units for each variant. Energetic differences between these two approaches are small, being up to 2.5 kcal/mol, deepening for B-Si complexes with heavier halogens. As can be seen from Table 5, the consequence of attaching the second HCN ligand is the reshaping of the electrophile, as manifested by the T-Tr-X-X angles, which are restored to values close to planarity. However, this geometry is not identical to that found in the isolated monomers because the alignment of the TX_3_ moiety versus the TrX_2_ group is similar to the situation in the dimer, not the monomer. Therefore, such arrangements are preferable for joining the second ligand, which is reflected in higher interaction energies for incorporating the second ligand. These higher energies correlate well with the bonding distances of the second ligand, which are shorter than those for the first HCN molecule, except for the two last trimers in Table 5. In complexes **13** and **22**, the interaction energy for the second ligand complexation is better despite the fact that the Tr⋯N contact is longer, which can be explained on the basis of the greater deformation that monomers **13** and **22** undergo through the complexation.

**Figure 7:**
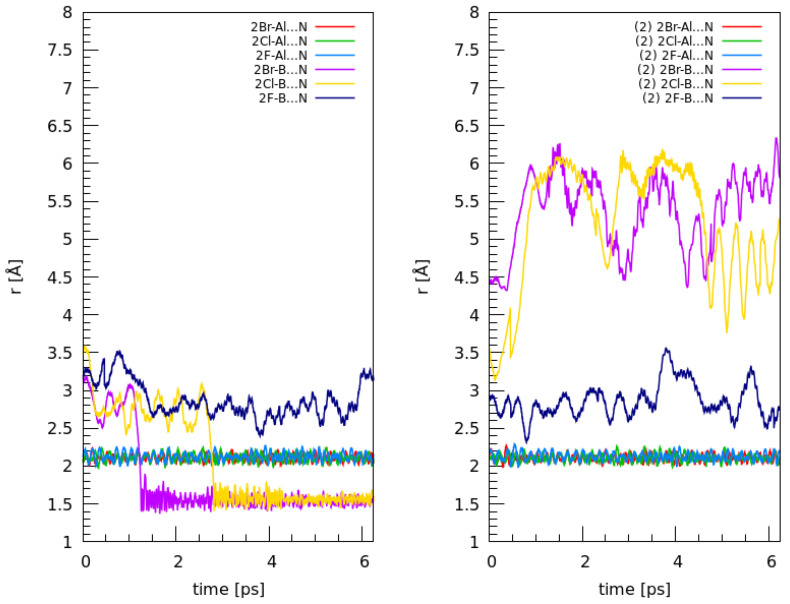
Time-evolution of metric parameters between the interacting pairs in the π-hole/π-hole trimers. **Left**: first interacting pair; **Right**: second interacting pair. Calculations performed at the PBE0-D3BJ/TZVP-MOLOPT-GTH level of the theory.

The nature of interactions in this group of trimers was additionally investigated by the BOMD computational setup. The results are presented in Figure 7, Figures S3 and S4 (angles for PBE0 only). In the case of PBE-D3BJ simulations, it can be seen that the behavior of the SiCl_3_-BCl_2_⋯NCH and SiBr_3_-BBr_2_⋯NCH interacting pairs changes significantly when compared to the results obtained for the dimers. In fact, for the second of the discussed complexes, the results obtained using the PBE-D3BJ indicate that there is no stable attractive interaction between any of the N…Tr atom pairs due to the too large distance between the π-hole donor and π-hole acceptor (which during the course of BOMD reaches values as large as 7.0 Å). Slightly different behavior characterized the SiCl_3_-BCl_2_⋯(NCH)_2_ complex, where there was no interaction between one of the N…Tr interacting pairs, whereas the other separation can be estimated as ca. 1.5 Å for most of the BOMD simulation time. In the case of the SiF_3_-BF_2_⋯(NCH)_2_ trimer, it can be noted that the N…Tr distances enlarged only by a small amount when compared to the dimer case—for ca. 0.1–0.2 Å. Thus, one can conclude that the interaction between both interacting pairs was maintained throughout the course of the BOMD simulation. The trimers with T = C, Tr = Al, and X = F, Cl, and Br behaved in a similar manner to the respective dimers—however, a negligible elongation of the N…Tr distance can be observed (ca. 0.1 Å). In these cases, the non-covalent interactions are stable and oscillate around 2.0 Å.

For the results obtained at the more rudimentary level of theory (PBE0-D3BJ/TZVP-MOLOPT-GTH), one can observe a different behavior of the SiBr_3_-BBr_2_⋯(NCH)_2_, where one of the N…Tr interacting pairs is stable and oscillates around 1.5 Å for most of the simulation time. However, noteworthy is the shortening of the separation between SiF_3_-BF_2_⋯NCH interacting pairs. Again, the outcome of the trimers with T = C, Tr = Al, and X = F, Cl, and Br is not affected at all when the exchange function is changed—these complexes were stable throughout the whole BOMD run. Inspecting the data gathered in Figure S4, we can see that the observed time-evolution of the Tr…N-C angles varies and strictly correlates with the distance between the interacting pairs—namely, the more pronounced the interaction is, the more linear the Tr…N-C arrangement (close to 180 degrees). Whereas every first interacting pair was characterized by a nearly linear arrangement of Tr…N-C throughout the whole simulation time (notable exceptions are SiF_3_-BF_2_⋯NCH and SiBr_3_-Bbr_2_⋯NCH interacting pairs), a different observation can be made when we consider (2) interacting pairs. In this case, every examined complex with T = Si and Tr = B took Tr…N-C angle values that deviated significantly from the ideal for the interaction strength (which agrees with conclusions drawn from the time-evolution of the distances (Figure 7)).

#### 2.3.2. “Unusual Trimers” and π-Hole/σ-Hole Trimers

According to the MEP results for dimers put in Table 4, one would expect to obtain three “unusual trimers” for **2**, **11**, and **20** initial dimers, but we have only managed to receive two such entities at MP2/aug-cc-pVDZ level: with CF_3_-AlF_2_ and CCl_3_-AlCl_2_ Lewis acids (**2** and **11**). The trimer of **20** + **2** HCN did not produce a stable minimum. These trimers are characterized by joining the second HCN ligand into the residual π-hole at a Tr⋯N-C angle of 158.4 and 164.4° along with Tr⋯N distances of 2.457 and 2.390 Å, for **2** and **11** complexes, respectively. The shorter distance and attack angle closer to linearity for complex **11** have resulted in interaction energy measured for the dimer⋯NCH construct being more favorable for this entity (−8.07 kcal/mol) by 0.45 kcal/mol than for complex **2**. The distances mentioned were longer by about 0.4 Å than the complexation distances found for the first HCN ligand in the dimer state. This difference is also seen in interaction energies since the energy of linking the second HCN molecule to the dimer is roughly four times lower than that gained for attaching the first ligand to the bare Lewis acid molecule. It points to some degree of anti-cooperativity between these two triel bonds.

**Figure 8:**
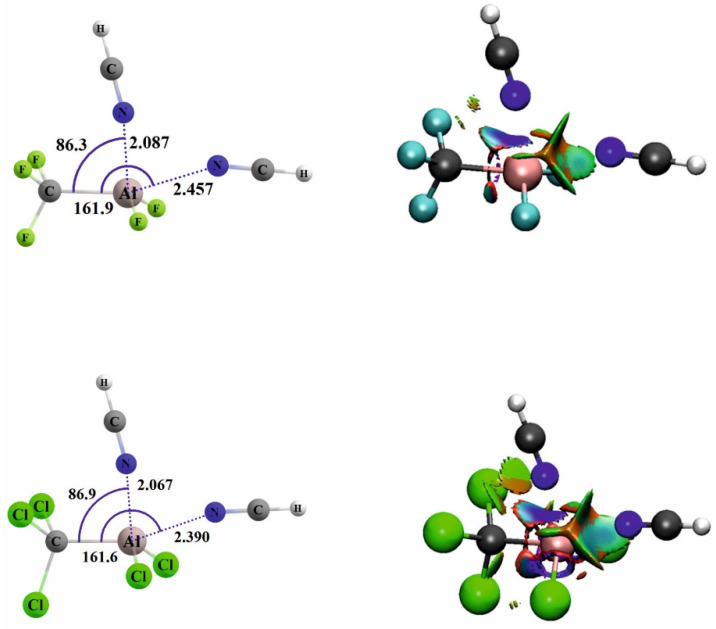
Optimized structures of “unusual trimers” obtained by attaching a second HCN molecule to the residual π-hole on the CX_3_-AlX_2_⋯NCH dimers, along with NCI molecular diagrams. The color code in NCI diagrams is as follows: Blue and green spheres correspond to noncovalent interaction regions (blue epitomizes stronger interaction), whereas brown and red ones correspond to weaker and stronger repulsive forces, respectively.

The optimized structures of these constructs, along with the NCI plots, are placed together in Figure 8, while QTAIM diagrams are displayed in Figure S5. Within the QTAIM methodology, the indicator of interaction is represented by the bond paths (dashed lines) and associated bond critical points (BCPs, green dots). For complex **2**, the bond path connecting HCN to the monomer via the original π-hole is observed, but there is no such pointer between the second HCN ligand and the Al atom. The expected interaction is altered by three other bond paths, namely those referring to the N⋯N and two N⋯F atom pairs. The electron density (ρ) at BCPs of these surprising interactions is nearly twice as small as ρ for a triel bond. The situation changes in trimer **11**, where, beside the same connections as in the previous complex, the missing triel bond path appears. The value of ρ at BCP for this unusual triel bond is 0.021 au, while ρ at BCP for the primary triel bond is 0.040 au. According to the potential energy density parameter (V) given by AIM analysis, one can approximate the bond energy [66]. In the current complex, the bond energies are 17 and 7 kcal/mol for the classic and unusual triel bonds, respectively. The significant difference in magnitude between these two bonds can also be quantified by the |V|/G ratio, where G is the kinetic energy density. A value of the |V|/G fraction higher than 1 indicates a high degree of covalent character in a given interaction, while a value less than 1 refers to a closed-shell interaction [67]. In the discussed trimer, this value for a stronger triel bond is 1.08, while for a weaker one, it is only 0.78. This evidence clearly demonstrates that in the trimer, the initial triel bond dominates over the second one, driven by the new hole.

The NCI formalism based on the correlation between reduced density gradient (RDG) and sign(λ_2_)ρ enables assigning qualitatively the real spaces to noncovalent interaction regions. A color-coded transformation of this procedure classifies the strong noncovalent regions as blue circled blots; average and weak regions are signified by green spheres; and finally, regions of steric repulsion are embodied by brown to red shapes. For both **2** and **11** trimers, the obtained picture is similar. The triel bond through the standard π-hole is evident by blue regions, which embody its covalent-like strength, while the secondary triel bond is signalized by green fragments, indicating its weaker magnitude.

The “unusual trimers” were further inspected with DLPNO-CCSD(T) and symmetry-adapted perturbation theory (SAPT) approaches. The complex (**2**) of CF_3_-AlF_2_ with two HCN molecules at its MP2/aug-cc-pVDZ geometry was selected for this purpose, and two series of distorted structures were generated as described in Section 3—the HCN molecules were displaced along a circular arc and served as interaction probes. The first series, labeled below “single HCN”, contains in fact dimeric, not trimeric, structures, while the second series, “(HCN)_2_”, are actual trimers treated however as (CF_3_-AlF_2_-HCN)⋯HCN dimers. Before, however, the scans with the HCN molecules are described, we note that the interaction energy between the CF_3_-AlF_2_ and the two HCN molecules at the optimized geometry amounts to −45.43 kcal/mol at the DLPNO-CCSD(T)/aug-cc-pVTZ level. When only the “vertical” HCN molecule (interacting with the π-hole site) is taken into account, the interaction energy is −36.01 kcal/mol, and the “horizontal” HCN molecule (at the σ-hole site) yields −17.35 kcal/mol. The two latter values sum to a value larger in magnitude than the first reported energy, and it is evident that there is anti-cooperativity between the two sites, as already suggested in the earlier discussion.

**Figure 9:**
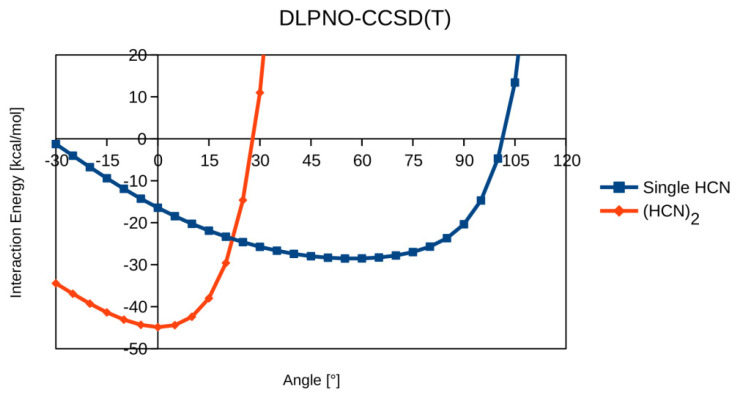
DLPNO-CCSD(T)/aug-cc-pVTZ interaction energies between the CF_3_-AlF_2_ molecule and the HCN or (HCN)_2_ molecules during a structural scan using one of the HCN molecules as the interaction probe.

Figure 9 shows the DLPNO-CCSD(T)/aug-cc-pVTZ interaction energy for the scan in which the HCN molecule at the σ-hole is displaced from its optimized site (labeled as 0°). When only a single HCN molecule is present, the σ-hole site is seemingly not attractive, while the π-hole region of attraction is very broad; its minimum (−28.5 kcal/mol) is at 55–60°. The difference between the interaction energy at the optimized structure (−36.01 kcal/mol, see above) is due to the nature of the scan, in which the molecule was kept in a linear arrangement with respect to the aluminum site. In the optimized structure, the reference angle (N-Al-N) is slightly larger, 75.6°. When the HCN molecule at the π-hole site is present, the situation is dramatically changed, and the HCN “probe” finds exactly the spot of the σ-hole at 0°. The origin of this behavior is revealed when the individual components of the interaction energy are studied—the SAPT approach was employed for this purpose.

**Figure 10:**
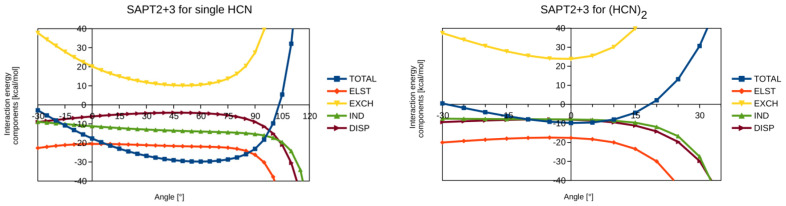
Symmetry-adapted interaction Energy decomposition at the SAPT2+3/aug-cc-pVTZ level for interaction between the CF_3_-AlF_2_ molecule and the HCN interaction probe—without (**left**) or with (**right**) inclusion of the HCN molecule at the π-hole. Labels: TOTAL—SAPT2+3 interaction energy; ELST—electrostatic term; EXCH—Pauli exchange repulsion; IND—induction (polarization) term; DISP—dispersion component.

The SAPT study at the SAPT2+3/aug-cc-pVTZ level yields ca. 6 kcal/mol weaker individual interactions than the DLPNO-CCSD(T) level (see Figure 9), but the locations of the minima are the same. The presence of the second HCN molecule at the π-hole site results in a much narrower range of angles at which the perturbative expansion is not divergent. The divergence in both cases is caused by orbital overlap—for single HCN, the overlap with the CF_3_ moiety fluorine atoms, while for the (HCN)_2_ case, the HCN molecules are the source of divergence. Electrostatic, induction, and dispersion terms in the non-divergent range are not much dependent on the scanned angle, and it is the sudden growth of the Pauli repulsion that makes the interaction repulsive at larger angle values. Figure 10 shows that the course of the total SAPT2+3 energy and the exchange term are very similar. This can be summarized by saying that the presence of the HCN molecule at the π-hole site creates an “exclusion zone” at scan angles larger than 15°, and this effect allows the second HCN molecule to find the σ-hole site.

The attempt to attach HCN to the σ-hole at the tetrel atom (σ-π trimers) was successful only for three dimers: **4**, **11**, and **22**, which is consistent with the σ-hole intensities at the tetrel end in dyads collected in Table S5. Only for the aforementioned structures were the values of σ-holes in dimeric forms positive, which allowed the association of such trimers, which are displayed in Figure S6 along with their interaction energies, which are all below 1 kcal/mol.

## 3. Materials and Methods

Full optimization of isolated TX_3_-TrX_2_ (Tr = B, Al, Ga; T = C, Si, Ge) monomers, dimers with Lewis bases (HCN, ammonia, and cyanide anion), as well as trimers with two hydrocyanic acids in the gas phase, was performed at the MP2/aug-cc-pVDZ level of theory [68,69,70] using the Gaussian 16 (Rev. C.01) set of codes [71]. This level of theory was confirmed as suitable for similar studies of noncovalent interactions [72,73,74,75].

The harmonic frequency analysis of normal modes allowed us to verify the existence of true minima on the potential energy surfaces. The interaction energy (E_int_) of each complex was computed as the difference in total electronic energy between the fully optimized complex and its subunits in the geometries adopted within the complex. The basis set superposition error (BSSE) was removed through the counterpoise procedure introduced by Boys and Bernardi [76]. The MEP (molecular electrostatic potential) analysis of monomers and dimers served to indicate the potential extrema at the 0.001 au electronic isodensity contour via the MultiWFN 3.7 software [77,78]. MEP visualization was assured by using the VMD 1.9.3 software [79]. Access to the Cambridge Structural Database (CSD, ver. 5.42) [59] was provided through the CCDC group of software [80,81].

The detection of unexpected “residual π-hole” trimers was followed by an additional investigation of one of these structures (CF_3_-AlF_2_ + 2HCN). One of the HCN molecules was used as an interaction probe, and a set of structures was generated by taking the optimized position of the nitrogen atom as an origin, rotating the Al-N vector towards or away from the first HCN molecule in 5-degree increments, and constructing the HCN probe molecule so that the Al-N-C-H arrangement was linear, but the bond distances were kept at the optimized values from the original trimer—see Figure S7 in the SI. This was carried out for the trimer (considered then as an “extended dimer” formed by two moieties, CF_3_-AlF_2_…HCN and the HCN probe) and the dimer (CF_3_-AlF_2_ and the HCN probe). For each of the generated structures, the interaction energy was calculated at the DLPNO-CCSD(T)/aug-cc-pVTZ level [82,83] using the ORCA 5.0.3 software [84]. In addition, the interaction energy decomposition was carried out within the Symmetry-Adapted Perturbation Theory (SAPT) framework [85] at the SAPT2+3/aug-cc-pVTZ level [86] using the PSI4 1.6.1. program [87].

In the last part of the computational study, the optimized structures of the dimers and trimers were taken as a starting point for Born-Oppenheimer molecular dynamics (BOMD) [88]. The simulations were performed in the NVE ensemble with the initial temperature set to 100 K using the PBE and PBE0 exchange-correlation functionals with the D3-BJ dispersion corrections [89,90,91]. Molecular orbitals were expanded using TZVP-MOLOPT-GTH (optimized for PBE and PBE0, respectively) basis sets [92]. The corresponding Goedecker–Teter–Hutter (GTH) pseudopotentials (optimized for PBE and PBE0) were used to represent the core electrons [93,94]. The calculations of the Hartree-Fock exact exchange were accelerated using the auxiliary density matrix method (ADMM), with FIT3 chosen as the auxiliary basis set [95]. The electron density cutoff was set to 1000 and 900 Ry for PBE and PBE0, respectively. The wavelet method was chosen to solve the Poisson equation, and the time step was set to 0.5 fs [96]. The simulations were carried out in cubic boxes with a = 16 Å (dimers) and a = 18 Å (trimers). The trajectory was collected for ca. 11 ps and 7 ps, respectively. The initial 1 ps of the trajectory was taken as an equilibration phase. The CP2K 8.2 program was used for the BOMD simulations [97]. Bader’s QTAIM methodology, encoded within the AIMAll suite of programs, was implemented to elucidate bond paths and determine their topological properties [98].

## 4. Conclusions

The set of 27 monomers of the general formula TX_3_-TrX_2_ (T = C, Si, Ge; Tr = B, Al, Ga; X = F, Cl, Br) molecules has been studied by means of quantum chemical methods. According to the MEP protocol, the π-hole spots were more intense than their σ-hole equivalents. The molecules possessing the most- (CX_3_-AlX_2_, 112 kcal/mol) and least-intense (SiX_3_-BX_2_, 27 kcal/mol) π-holes were selected to study their dimerization abilities with hydrogen cyanide. The wide span of interaction energies of the obtained dimers (from −6.5 to roughly −30 kcal/mol) correlated with the MEP of monomers for 5 out of 6 complexes; however, for the SiBr_3_-BBr_2_⋯HCN dimer, we have found an anomaly. Despite the lowest value of the π-hole magnitude at the B atom in the SiBr_3_-BBr_2_ monomer, its complex with NCH had a surprisingly high interaction energy (−28.72 kcal/mol), similar to the ones achieved for complexes with the strongest π-holes predicted. The postulated explanation is the considerable deformation of the SiBr_3_-BBr_2_, which made this molecule more susceptible to the incoming nucleophile. Indeed, the deformation energy for this complex (16.6 kcal/mol) was firmly larger than in the case of the remaining dyads. In the case of the CX_3_-AlX_2_⋯HCN dimers featuring high π-hole potential, an interesting discovery was finding the new maximum of MEP near the region of already incorporated HCN ligand, which was baptized as the “residual π-hole.” Another HCN entity was able to attach to this maximum, thereby making an “unusual trimer” in which two Lewis bases occupied the primary π-hole region. This situation was not observed for ligands with more intense negative potential, such as ammonia or cyanide anion. Therefore, based on our results, one can claim that the conditions for raising the residual π-hole are the massive depth of the original π-hole and incorporating Lewis bases of moderate or weak negative potential. The BOMD results for selected structures showed their stability and enabled the discussion of the strength of interactions as a function of time. On the basis of the BOMD, we could follow structural changes that caused shifts in electron density and thus see changes in π holes. The energy decomposition based on the SAPT method allowed the qualitative analysis of the shares of individual energies while the geometry of the studied complexes varied.

## Figures and Tables

**Table 1:** MEP maxima of π-holes at the 0.001 au isodensity contour on the most stable conformers of isolated TX_3_-TrX_2_ monomers (Tr = B, Al, Ga; T = C, Si, Ge; X = F, Cl, Br). The least and most intense maxima for each X atom group are highlighted in orange. Data given in kcal/mol.

No.	V_s,max_	No.	V_s,max_	No.	V_s,max_
X = F		X = Cl		X = Br	
**1**	63.3	**10**	38.3	**19**	29.8
**2**	111.8	**11**	84.2	**20**	70.7
**3**	94.6	**12**	74.1	**21**	64.5
**4**	57.0	**13**	35.0	**22**	26.7
**5**	98.1	**14**	76.7	**23**	67.8
**6**	85.5	**15**	67.6	**24**	59.7
**7**	63.1	**16**	38.5	**25**	29.9
**8**	107.4	**17**	81.8	**26**	71.6
**9**	93.5	**18**	72.1	**27**	62.9

**Table 2:** Selected geometric parameters (Å, degrees) and interaction energies (kcal/mol) in π-hole complexes between selected monomers and HCN (gas phase, with BSSE correction).

	V_s,max_^a^	R(N⋯Tr)	∡ (Tr⋯N-C)	∡ (T-Tr⋯N)	∡ (T-Tr-X-X)	E_int_	E_bin_	E_def_
**2** CF_3_-AlF_2_⋯HCN	111.8	2.067	176.1	94.4	150.8	−29.59	−25.08	4.51
**11** CCl_3_-AlCl_2_⋯HCN	84.2	2.054	177.1	94.8	147.8	−28.65	−23.92	4.73
**20** CBr_3_-AlBr_2_⋯HCN	70.7	2.056	177.5	95.3	146.6	−27.48	−22.63	4.85
								
**4** SiF_3_-BF_2_⋯HCN	57.0	2.471	175.7	85.1	171.8	−7.20	−6.65	0.55
**13** SiCl_3_-BCl_2_⋯HCN	35.0	2.507	174.0	84.4	167.1	−6.50	−5.78	0.72
**22** SiBr_3_-BBr_2_⋯HCN	26.7	1.572	174.6	104.0	128.3	−28.72	−12.15	16.57

^a^ this is the value of π-hole magnitude obtained for the respective monomer.

**Table 3:** Selected geometric parameters and interaction energies in π-hole complexes between selected monomers and NH_3_ (gas phase, with BSSE correction).

	Dimers with NH_3_					Dimers with CN^−^				
	R(N⋯Tr)	∡ (T-Tr-X-X)	E_int_	E_bin_	E_def_	R(C⋯Tr)	∡ (T-Tr-X-X)	E_int_	E_bin_	E_def_
**2**	2.034	148.4	−46.11	−39.92	6.19	2.009	120.2	−104.14	−84.11	20.03
**11**	2.023	144.1	−45.86	−39.34	6.52	1.990	119.8	−104.59	−85.04	19.55
**20**	2.035	141.8	−44.57	−37.62	6.95	1.987	120.1	−103.02	−84.17	18.85
										
**4**	1.659	132.6	−42.95	−23.38	19.57	1.625	118.2	−101.22	−65.98	35.24
**13**	1.623	129.2	−50.70	−30.98	19.72	1.585	117.1	−112.86	−81.62	31.24
**22**	1.615	127.6	−52.52	−33.37	19.15	1.574	116.9	−115.40	−86.64	28.76

**Table 4:** MEP maxima of π-holes at the 0.001 au isodensity contour on the selected TX_3_-TrX_2_ dimers (Tr = B, Al, Ga; T = C, Si, Ge; X = Cl, F, H) with hydrogen cyanide, ammonia, and cyanide anion. Data given in kcal/mol.

Monomer	π-Hole Maxima for Dimers with:		
	NCH	NH_3_	CN^−^
**2**	59.0 (23.1) ^a^	27.5	−78.7
**11**	25.9 (25.3)	56.4	−66.6
**20**	12.7 (22.6)	12.7	−62.4
			
**4**	37.7	−2.8	-
**13**	16.0	-	−84.4
**22**	−11.4	−6.9	−67.2

^a^ the values of V_s,max_ in residual π-hole are given in parentheses.

**Table 5:** Selected geometric parameters and interaction energies in π-hole complexes between selected monomers and 2 HCN molecules. Distances in Å, angles in degrees, energies in kcal/mol.

Trimer with HCN	V_s,max_^a^	R(N⋯Tr)	∡ (T-Tr⋯N)	∡ (T-Tr-X-X)	E_int_	E_bin_	E_def_
**2** CF_3_-AlF_2_⋯(HCN)_2_	59.0	2.161	86.5	−177.9	−17.38 ^b^	−11.09	6.29
		2.155	91.1		−17.46 ^c^	−12.52	4.94
**11** CCl_3_-AlCl_2_⋯(HCN)_2_	25.9	2.151	87.0	−176.9	−16.23	−7.21	9.02
		2.146	94.3		−17.22	−11.88	5.34
**20** CBr_3_-AlBr_2_⋯(HCN)_2_	12.7	2.155	87.4	−176.7	−14.90	−5.06	9.84
		2.151	95.4		−16.24	−6.39	9.85
**4** SiF_3_-BF_2_⋯(HCN)_2_	37.7	2.675	78.5	−173.8	−4.34	−0.45	3.89
		2.565	92.5		−4.84	−0.78	4.06
**13** SiCl_3_-BCl_2_⋯(HCN)_2_	16.0	2.695	80.4	−178.5	−2.59	2.77	5.36
		2.854	94.9		−4.66	8.38	13.04
**22** SiBr_3_-BBr_2_⋯(HCN)_2_	−11.4	2.638	81.4	176.2	−1.60	14.72	16.32
		3.090	98.2		−4.52	12.44	16.96

^a^ This is the value of the remaining π-hole magnitude obtained for the respective dimer. ^b^ Value for the first ligand. ^c^ Value for the second ligand.

## Data Availability

The data presented in this study are available in Supplementary Materials (Figures S1–S7 and Tables S1–S7).

## Supplementary Materials

The following supporting information can be downloaded at: https://www.mdpi.com/article/10.3390/ijms24097881/s1.
