# Supplementary material for: Static and Dynamical Quantum Studies of CX3-AlX2 and CSiX3-BX2 (X = F, Cl, Br) Complexes with Hydrocyanic Acid: Unusual Behavior of Strong π-Hole at Triel Center
Source: Int J Mol Sci. 2023 Apr 26;24(9):7881. doi: 10.3390/ijms24097881 (PMC10177972; doi:10.3390/ijms24097881)
Supplement: Supplementary file 1 [file ijms-24-07881-s001.zip › ijms-2340444-supplementary.pdf]

Supplementary Information

Static and Dynamical Quantum Studies of CX_3-AlX_2 and $CSiX_3$-BX_2 (X = F, Cl, Br) Complexes with Hydrocyanic Acid: Unusual Behavior of Strong π-Hole at Triel Center

Mariusz Michalczyk [1,*], Kamil Wojtkowiak [2], Jarosław J. Panek [2], Aneta Jezierska [2] and Wiktor Zierkiewicz [1,*]

[1] Faculty of Chemistry, Wrocław University of Science and Technology, Wybrzeże Wyspiańskiego 27,
50-370 Wrocław, Poland
[2] Faculty of Chemistry, University of Wrocław, ul. F. Joliot-Curie 14, 50-383 Wrocław, Poland;
kamil.wojtkowiak@chem.uni.wroc.pl (K.W.); jaroslaw.panek@chem.uni.wroc.pl (J.J.P.); aneta.jezierska@chem.uni.wroc.pl (A.J.)
* Correspondence: mariusz.michalczyk@pwr.edu.pl (M.M.); wiktor.zierkiewicz@pwr.edu.pl (W.Z.)

Table of contents:

1. **Figure S1.** Structures (including refcodes) of a crystalline compound from CSD database with a geometry corresponding to the one discussed in the work.
2. **Table S1.** MEP maxima at the 0.001 au isodensity contour on isolated TF_3-TrF_2 monomers (Tr= B, Al, Ga; T= C, Si, Ge). Data given in kcal/mol.
3. **Table S2.** MEP maxima at the 0.001 au isodensity contour on isolated TCl_3-$TrCl_2$ monomers (Tr= B, Al, Ga; T= C, Si, Ge). Data given in kcal/mol.
4. **Table S3.** MEP maxima at the 0.001 au isodensity contour on isolated TBr_3-$TrBr_2$ monomers (Tr= B, Al, Ga; T= C, Si, Ge). Data given in kcal/mol.
5. **Table S4.** Summary of trimers geometry optimization.
6. **Table S5.** The σ-hole maxima at the 0.001 au isodensity contour on TX_3-TrX_2 dimers (Tr= B, Al; T= C, Si; X= F, Cl, Br) with hydrogen cyanide. Data given in kcal/mol.
7. **Figure S2.** Time-evolution of metric parameters between the interacting pairs. Calculations performed at the PBE-D3BJ/TZVP-MOLOPT-GTH level of theory.
8. **Figure S3.** Time-evolution of metric parameters between the interacting pairs in the **π-hole/π-hole** trimers. Left: first interacting pair, Right: second interacting pair. Calculations performed at the PBE-D3BJ/TZVP-MOLOPT-GTH level of theory.

9. **Figure S4.** Time-evolution of Tr...N-C angles throughout the BOMD simulation for the **π-hole/π-hole** trimers. Calculations performed at the PBE0-D3BJ/TZVP-MOLOPT-GTH level of the theory.
10. **Figure S5.** QTAIM diagrams for "unusual trimers" studied in this work. QTAIM bond paths are represented by dashed lines while numbers given are ρ at BCPs in au.
11. **Figure S6.** Optimized structures of trimers obtained by attaching second HCN molecule to the σ-hole site of TX_3-TrX_2···NCH dimers.
12. **Figure S7**. Overview of structures generated for the interaction energy study of the "residual π-hole" CF_3-AlF_2 + 2HCN trimer. The optimized atomic positions are shown, but the HCN molecule at 0 degrees label was removed and used as an interaction probe. It was located at the positions indicated by coloured sticks and numbered with angle values from -30 to 120 degrees. The vertically aligned HCN molecule was removed in the first set of structures, but present in the second set.
13. **Table S6.** Coordinates of dimers
14. **Table S7.** "Unusual" trimers coordinates

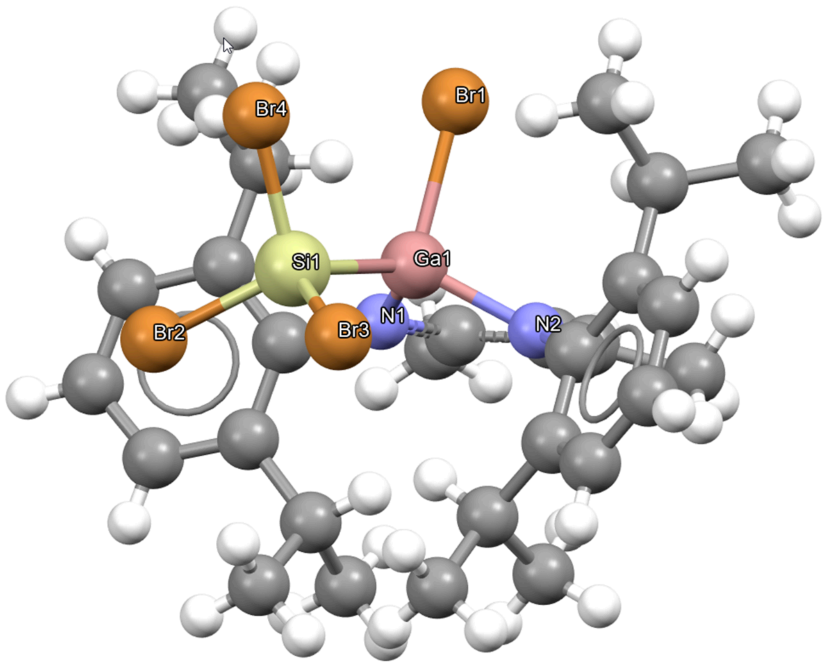

Database Identifier: HUHKUT

Formula: $[L(Br)Ga]_2Si$:–CO (L = HC[C(Me)N(2,6-^{i}Pr$_2$-C$_6$H$_3$)]$_2$)

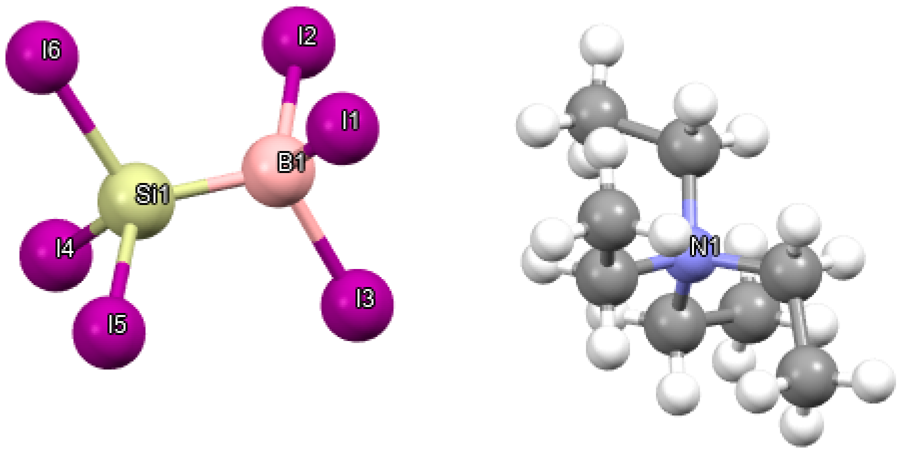

Database Identifier: JEKXUV

Formula: $[Et_4N][I_3SiBI_3]$

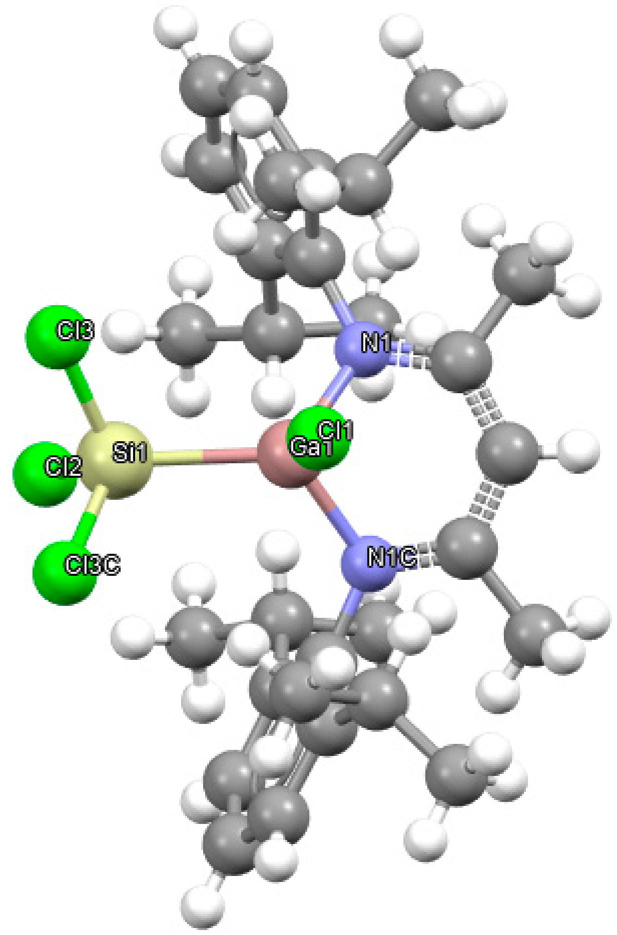

Database Identifier: JOHFAO

Formula: [Cl_3Si{ClGa(DDP)}], DDP= 2-{(2,6-diisopropyl-phenyl)amino}–4-{(2,6-diisopropylphenyl)imino}–2-pentene

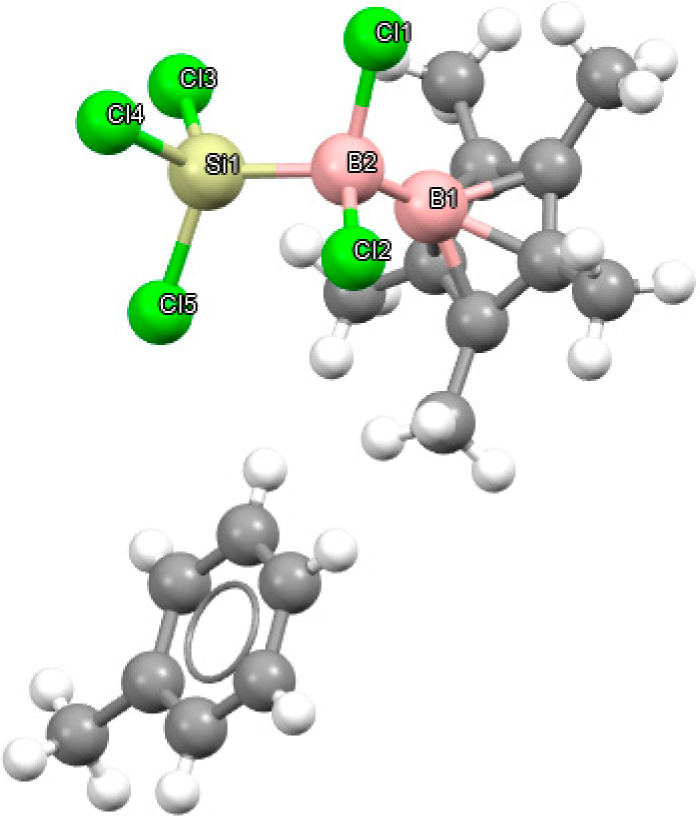

Database Identifier: WIZZEK

Formula: $(C_5Me_5)B \rightarrow BCl_2SiCl_3$

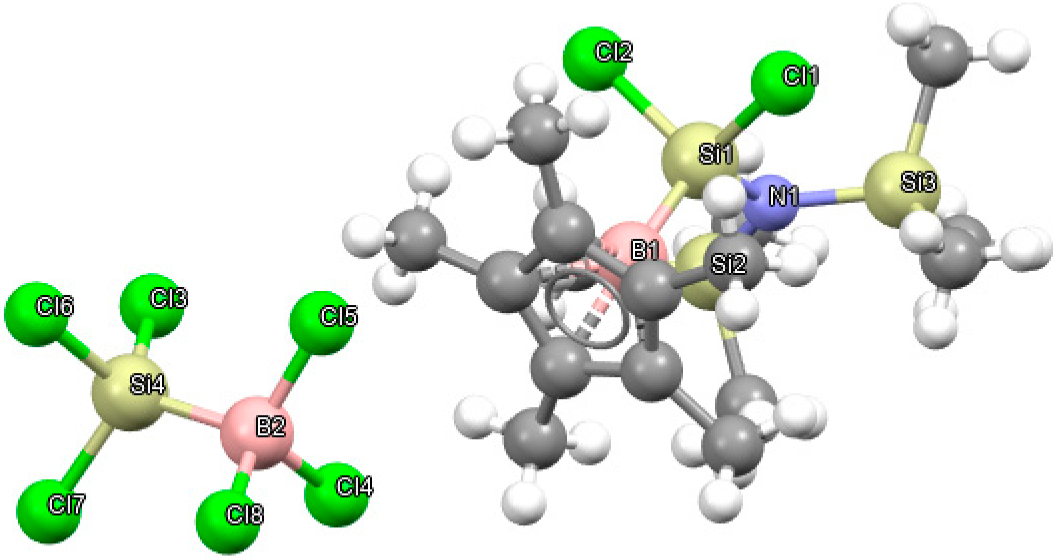

Database Identifier: AWAWON

Formula: $[Cp^*BSi(Cl)_2N(TMS)_2)][BCl_3SiCl_3]$

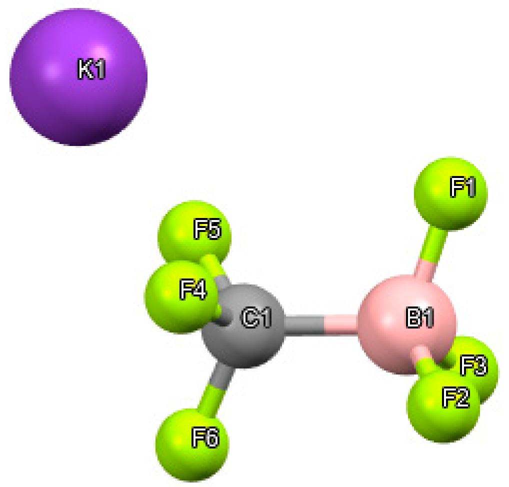

Database Identifier: KTFMFB

Formula: $K[CF_3BF_3]$

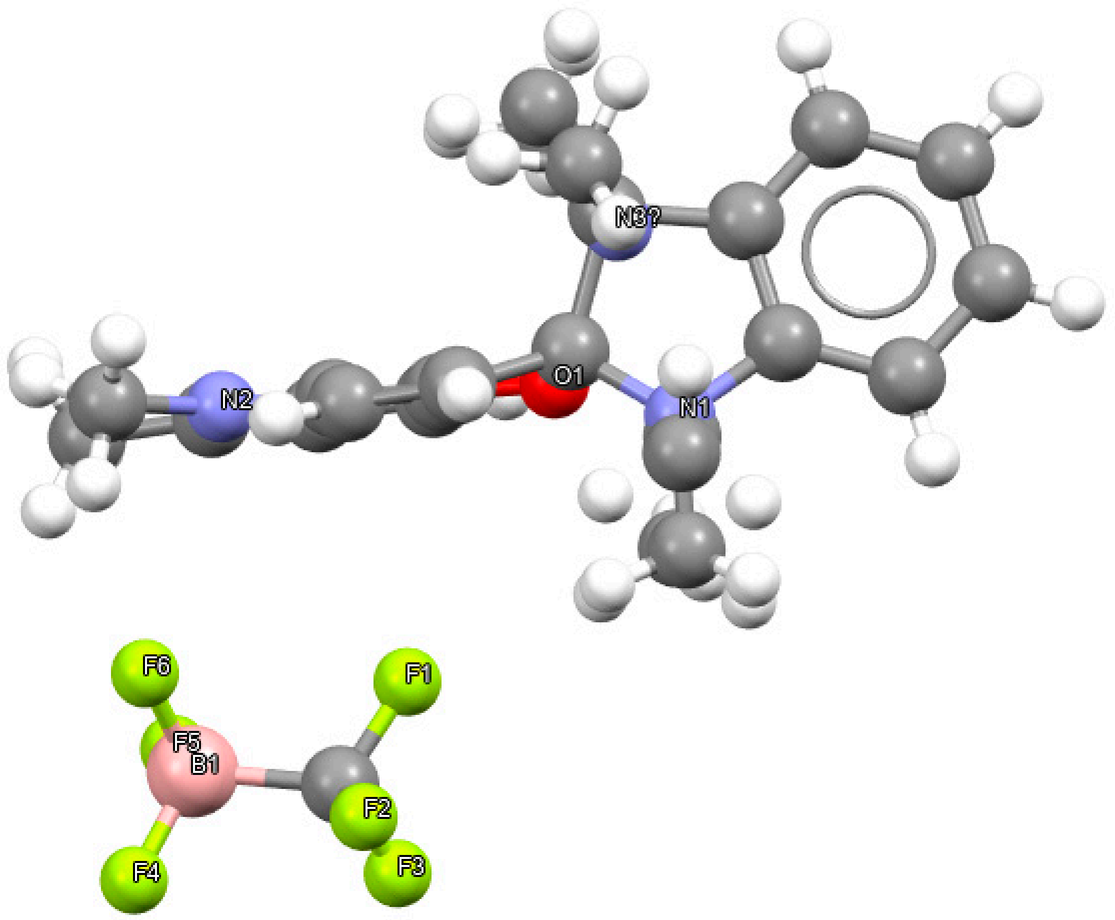

Database Identifier: MOVNIX

Formula: [**PSP**]X; X = I, NO_3, SCN, BF_4, ClO_4, PF_6, CF_3BF_3, OTf, and BPh_4, PSP= 1-methyl-3,3,5′,6′-tetramethylspiro[indoline-2,2′-[2*H*]pyran[3,2-*b*]pyridinium]

Figure S1. Structures (including refcodes) of a crystalline compound from CSD database with a geometry corresponding to the one discussed in the work.

Table S1. MEP maxima at the 0.001 au isodensity contour on isolated TF_3-TrF_2 monomers (Tr= B, Al, Ga; T= C, Si, Ge). Data given in kcal/mol.

	B	Al	Ga
C	σ 18.9/12.3*<br>π 63.3	σ 30.0/7.9*<br>π 111.8	σ 35.5/15.0*<br>π 94.6
Si	σ 51.5/36.3*<br>π 57.0	σ 42.9/32.8*<br>π 98.1	σ 48.8/42.3*<br>π 85.5
Ge	σ 55.4/33.4*<br>π 63.1	σ 47.4/29.0*<br>π 107.4	σ 53.1/38.3*<br>π 93.5

*Maximum lying on the extension of the Tr-T bond (at T atom)

Table S2. MEP maxima at the 0.001 au isodensity contour on isolated TCl_3-$TrCl_2$ monomers (Tr= B, Al, Ga; T= C, Si, Ge). Data given in kcal/mol.

	B	Al	Ga
C	σ 1.0/1.7*<br>π 38.3/19.0	σ 11.0/4.1*<br>π 84.2/60.8	σ 5.3*<br>π 74.1/60.8
Si	σ 31.2/16.7*<br>π 35.0	σ 25.7/17.0*<br>π 76.7	σ 28.0/22.1*<br>π 67.6
Ge	σ 36.1/16.0*<br>π 38.5	σ 29.7/15.6*<br>π 81.8	σ 31.7/20.7*<br>π 72.1

*Maximum lying on the extension of the Tr-T bond (at T atom)

Table S3. MEP maxima at the 0.001 au isodensity contour on isolated TBr_3-$TrBr_2$ monomers (Tr= B, Al, Ga; T= C, Si, Ge). Data given in kcal/mol.

	B	Al	Ga
C	σ -1.7/1.3*<br>π 29.8/10.4	σ 4.1/5.8*<br>π 70.7/40.6	σ 6.6/5.2*<br>π 64.5/42.9
Si	σ 25.0/11.5*<br>π 26.7	σ 16.6/13.1*<br>π 67.8	σ 17.8/17.0*<br>π 59.7
Ge	σ 28.7/11.6*<br>π 29.9	σ 19.9/12.0*<br>π 71.6	σ 21.2/15.9*<br>π 62.9

*Maximum lying on the extension of the Tr-T bond (at T atom)

Table S4. Summary of trimers geometry optimization.

**TX_3-TrX_2 + 2 NCH**	Unusual trimer (diagonally aligned)	Trimer π-π	Trimer σ-π
**2**	√	√	-
**11**	√	√	-
**20**	-	√	-
**4**	-	√	√
**13**	-	√	√
**22**	-	√	√

Table S5. The σ-hole maxima at the 0.001 au isodensity contour on TX_3-TrX_2 dimers (Tr= B, Al; T= C, Si; X= F, Cl, Br) with hydrogen cyanide. Data given in kcal/mol.

	**2** CF_3-AlF_2	**11** CCl_3-$AlCl_2$	**20** CBr_3-$AlBr_2$	**4** SiF_3-BF_2	**13** $SiCl_3$-BCl_2	**22** $SiBr_3$-BBr_2
σ-hole maxima	-7.6	-7.5	-5.3	33.8	9.5	3.9

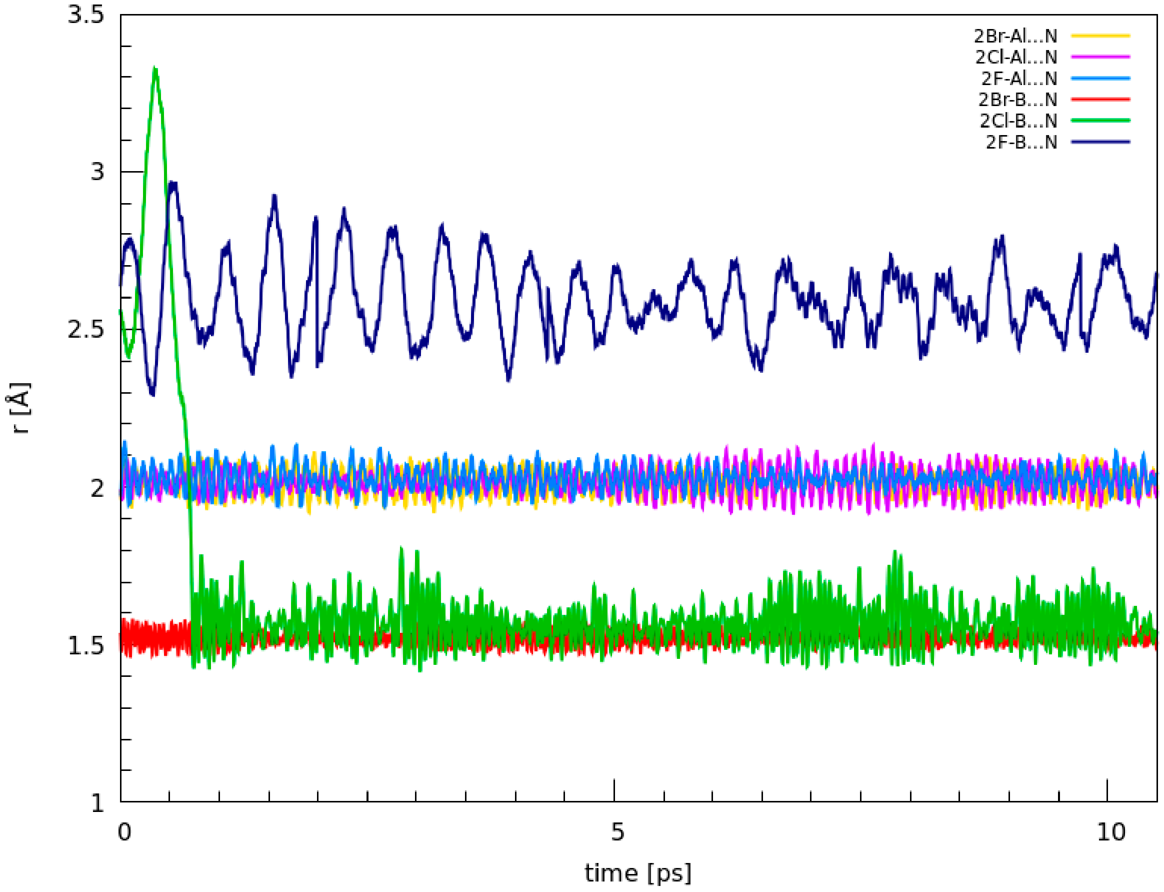

Figure S2. Time-evolution of metric parameters between the interacting pairs. Calculations performed at the PBE-D3BJ/TZVP-MOLOPT-GTH level of theory.

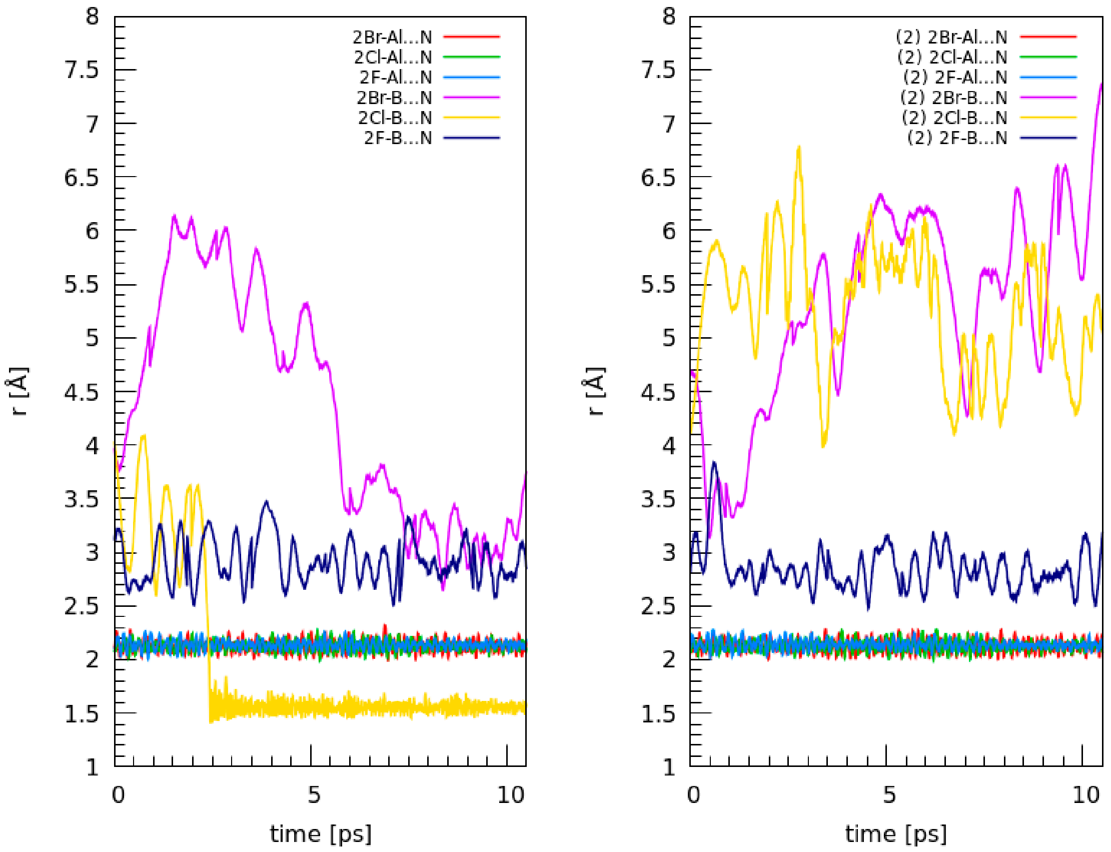

Figure S3. Time-evolution of metric parameters between the interacting pairs in the **π-hole/π-hole** trimers. Left: first interacting pair, Right: second interacting pair. Calculations performed at the PBE-D3BJ/TZVP-MOLOPT-GTH level of theory.

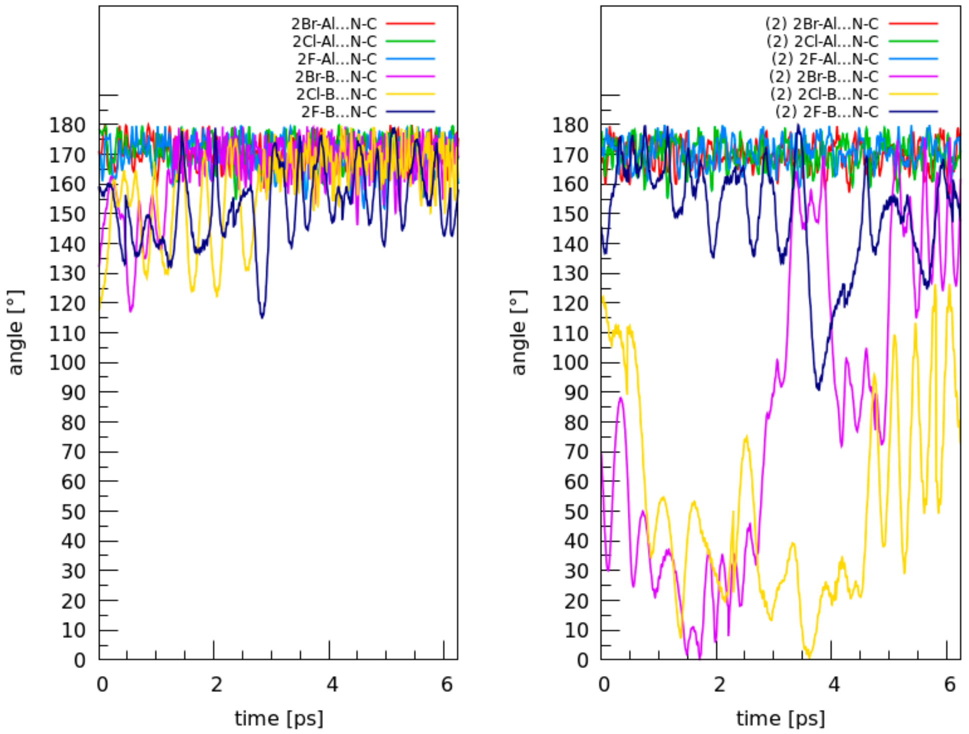

Figure S4. Time-evolution of Tr...N-C angles throughout the BOMD simulation for the **π-hole/π-hole** trimers. Calculations performed at the PBE0-D3BJ/TZVP-MOLOPT-GTH level of the theory.

CF_3-AlF_2 (**2**) + 2 HCN	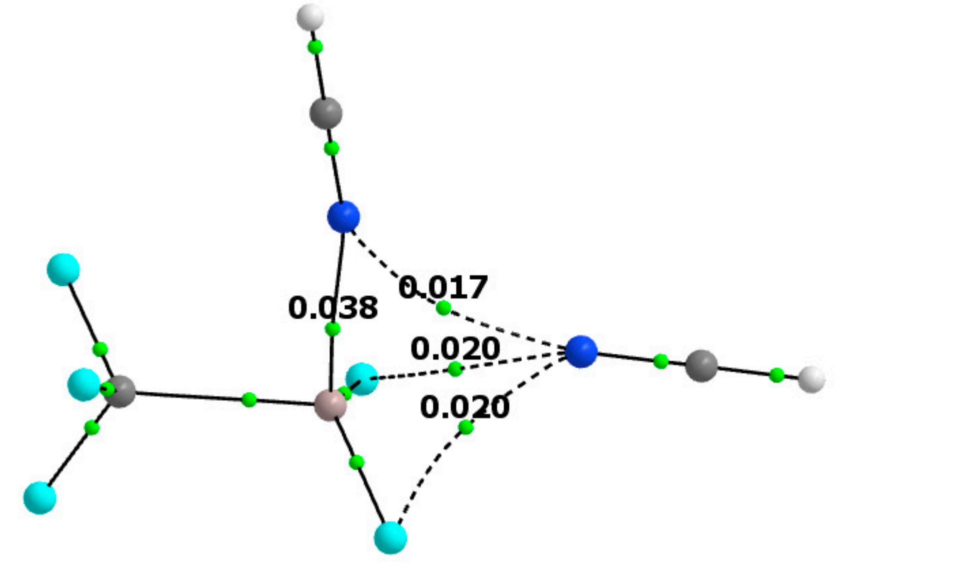
CCl_3-$AlCl_2$ (**11**) + 2 HCN	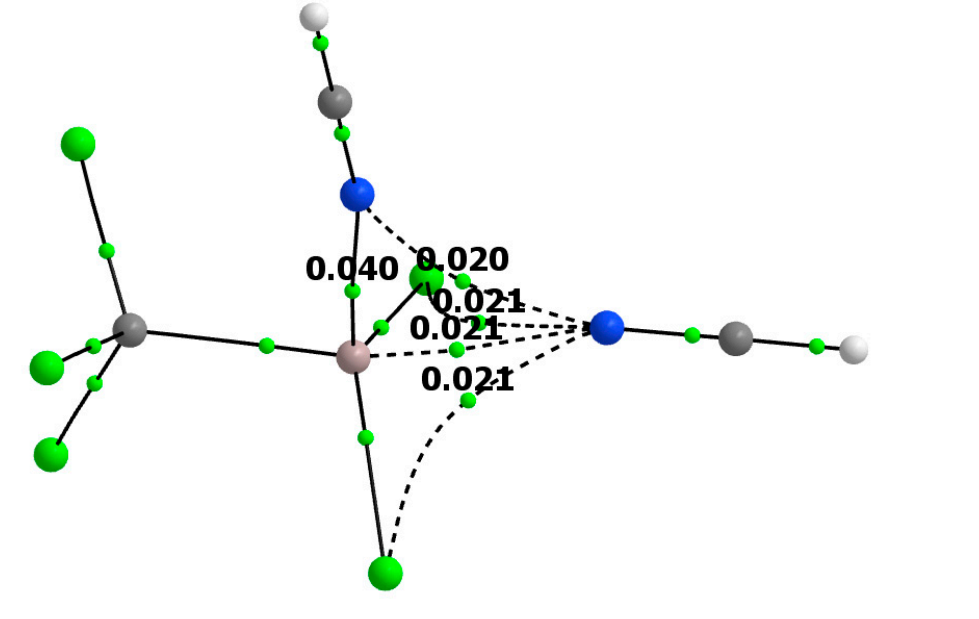

Figure S5. QTAIM diagrams for "unusual trimers" studied in this work. QTAIM bond paths are represented by dashed lines while numbers given are ρ at BCPs in au.

**TX_3-TrX_2 + 2 HCN**	Trimers σ-π	E_{int}
SiF_3-BF_2 **(4)**	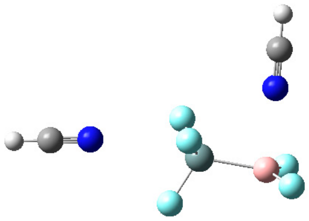	-0.55
$SiCl_3$-BCl_2 (**13**)	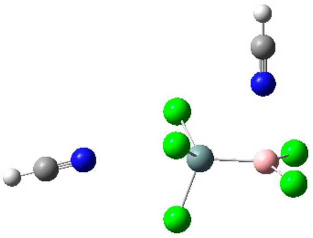	-0.66
$SiBr_3$-BBr_2 (**22**)	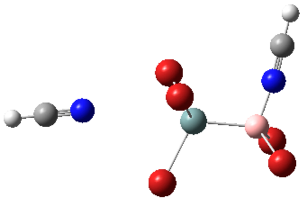	-0.60

Figure S6. Optimized structures of trimers obtained by attaching second HCN molecule to the σ-hole site of TX_3-TrX_2···NCH dimers.

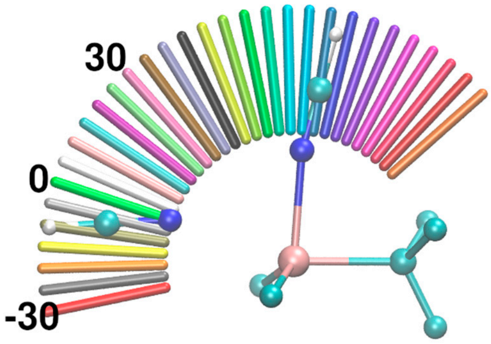

Figure S7. Overview of structures generated for the interaction energy study of the "residual π-hole" CF_3-AlF_2 + 2HCN trimer. The optimized atomic positions are shown, but the HCN molecule at 0 degrees label was removed and used as an interaction probe. It was located at the positions indicated by coloured sticks and numbered with angle values from -30 to 120 degrees. The vertically aligned HCN molecule was removed in the first set of structures, but present in the second set.

Table S6. Coordinates of dimers.

Dimer with HCN	coords			
CF_3-AlF_2	C	0.27737400	-0.04461800	-0.35622200
	Al	1.05524600	-1.91431500	-0.24479300
	F	-0.88138500	0.10924400	-1.07174100
	F	1.18514200	0.84335500	-0.91221800
	F	0.01187800	0.46318300	0.90604400
	F	1.84883200	-2.49374400	-1.61186100
	F	0.29243600	-2.99672900	0.79486500
	N	2.66796600	-1.34556000	0.91604400
	C	3.55291000	-0.94824400	1.57104300
	H	4.37004800	-0.58532200	2.17504600
SiF_3-BF_2	Si	0.29104900	-0.07528600	-0.42275500
	B	1.01989600	-1.95745400	-0.41778500
	F	-0.49028000	0.27417400	-1.81086400
	F	1.45976900	1.05233000	-0.25308100
	F	-0.76989100	0.17256600	0.79070300
	F	1.47168600	-2.52780000	-1.55177200
	F	1.22525100	-2.61696500	0.73906300
	N	-1.33729500	-2.64308300	-0.69705000
	C	-2.43454100	-3.05452100	-0.82697400
	H	-3.43733300	-3.43168300	-0.94592400
CCl_3-$AlCl_2$	C	0.09619300	-0.12018100	-0.43605500
	Al	0.67955700	-2.04374300	-0.43126400
	Cl	-0.85314100	0.24774800	-1.92885500
	Cl	1.53624100	0.95729100	-0.38165800
	Cl	-0.94770900	0.22639800	0.99754300
	Cl	1.50271400	-2.78235200	-2.25293100
	Cl	1.38354300	-2.80921000	1.42888600
	N	-1.22808700	-2.80304500	-0.49837900
	C	-2.33609500	-3.18105300	-0.53678200
	H	-3.35638300	-3.53023600	-0.57215700
$SiCl_3$-BCl_2	Si	0.26548400	-0.06425400	-0.32402100

	B	0.94744200	-1.96237400	-0.21039900
	Cl	-1.12724500	0.18730400	-1.84706700
	Cl	1.88182800	1.18775500	-0.72986400
	Cl	-0.59839800	0.57510500	1.45572100
	Cl	1.15685800	-2.89130600	-1.69180900
	Cl	1.63722100	-2.53989200	1.30348000
	N	-1.44612400	-2.55387800	0.24245900
	C	-2.53559600	-2.95032300	0.46452100
	H	-3.53149900	-3.30983600	0.66709100
CBr_3-$AlBr_2$	C	0.63503200	0.16112000	-0.37273600
	Al	1.33198000	-1.71812300	-0.39917200
	Br	-0.29631500	0.58043400	-2.04719300
	Br	2.16999500	1.35881200	-0.16272300
	Br	-0.60621000	0.42156300	1.12333300
	Br	2.40854200	-2.36632100	-2.30651300
	Br	2.02371200	-2.56404100	1.60655400
	N	-0.51131300	-2.60032900	-0.62454200
	C	-1.58315400	-3.05656900	-0.75128000
	H	-2.57020300	-3.47634000	-0.86819800
$SiBr_3$-BBr_2	Si	0.58386700	0.02596900	-0.48589900
	B	1.00077400	-1.95365300	-0.29539600
	Br	-0.85026100	0.35399900	-2.19560200
	Br	2.42793200	1.22697500	-0.83199200
	Br	-0.45914400	0.77987700	1.36634900
	Br	1.71885600	-2.70525800	-1.99230700
	Br	2.08339700	-2.30823500	1.33630600
	N	-0.40480900	-2.61811800	-0.06223900
	C	-1.49467300	-3.01183100	0.10397900
	H	-2.49476800	-3.38527300	0.25787800

Table S7. "Unusual" trimers coordinates

AIM	Coords			
CF_3-AlF_2 + 2 HCN	C	0.81486300	-0.77003100	-1.46206300
	Al	0.06431500	-1.68196000	0.20707400
	F	0.78442100	-1.60257700	-2.57949200
	F	2.10832200	-0.31450000	-1.38762900
	F	0.05925300	0.33649200	-1.84615200
	F	0.85947000	-3.16683200	0.38434400
	F	-0.14038900	-0.49278400	1.39555700
	N	-1.69677000	-1.92777300	-0.88514800
	C	-2.56325300	-1.95395200	-1.67319300
	H	-3.36298200	-1.97998100	-2.39559800
	N	-1.45764600	-2.83571900	1.75307900
	C	-1.82672600	-3.34946600	2.74644900
	H	-2.15732700	-3.81809400	3.65866500
CCl_3-$AlCl_2$ + 2 HCN	C	0.75175300	-0.78024600	-1.49374900
	Al	0.04037400	-1.68783900	0.19971900
	Cl	0.73587200	-1.90295300	-2.92107300
	Cl	2.44941800	-0.24691800	-1.22140600
	Cl	-0.23190400	0.67846500	-1.94388700
	Cl	1.18802000	-3.50258400	0.35953500
	Cl	-0.08611200	-0.09930700	1.64765600
	N	-1.73170000	-1.96304900	-0.82892300
	C	-2.63024500	-2.01619200	-1.57966800
	H	-3.45445900	-2.06302300	-2.27303200
	N	-1.40814100	-2.81065200	1.73406800
	C	-1.86593900	-3.34024800	2.68119500
	H	-2.27138400	-3.82262900	3.55545900
CBr_3-$AlBr_2$ + 2 HCN	C	0.68268500	-0.81461200	-1.47083500
	Al	-0.02264000	-1.71955800	0.22216200
	Br	0.68052400	-2.01612900	-3.00946300
	Br	2.50744300	-0.25579300	-1.13888200
	Br	-0.36125300	0.76125400	-1.95861100

	Br	1.26409700	-3.60724900	0.34716500
	Br	-0.09250300	0.01181900	1.71620400
	N	-1.76265200	-1.98646200	-0.79820300
	C	-2.61458000	-2.00975700	-1.58277000
	H	-3.39352500	-2.02630200	-2.31260700
	N	-1.35319500	-2.78839100	1.72952100
	C	-1.81902300	-3.31345700	2.65618400
	H	-2.22982500	-3.79253800	3.51602900
